# The two extremes of physiological tooth resorption in primary tooth with or without the permanent successor tooth

**DOI:** 10.1590/2177-6709.26.6.e21ins6

**Published:** 2021-12-15

**Authors:** Alberto CONSOLARO, Moacyr Tadeu RODRIGUES, Renata Bianco CONSOLARO, Giovana Gonçalves MARTINS

**Affiliations:** 1 Universidade de São Paulo, Faculdade de Odontologia de Bauru (Bauru/SP, Brazil).; 2 Universidade de São Paulo, Faculdade de Odontologia de Ribeirão Preto, Programa de Pós-graduação em Odontopediatria (Ribeirão Preto/SP, Brazil).; 3 Cirurgião bucomaxilofacial da Secretaria de Estado da Saúde (Porto Velho/RO, Brazil).; 4 Centro Universitário de Adamantina (Adamantina/SP, Brazil).

**Keywords:** Primary tooth, Rhizolysis, Alveolodental ankylosis, Replacement resorption

## Abstract

**Introduction:**

Assessment of two radiographic images reveals two distinct, extreme situations of physiological tooth resorption, characteristic of primary teeth with or without permanent successor, due to partial anodontia.

**Discussion:**

In all primary teeth, rhizolysis begins after the completion of formation, thanks to the apoptosis of their cells. When apoptosis induced by cementoblasts has denuded the root of these cells, the process of rhizolysis inevitably begins: This will be accelerated by mediators arising from the pericoronal follicle. When there is no permanent successor due to partial anodontia, rhizolysis occurs extremely slowly, and months later, without the epithelial rests of Malassez that were dead due to apoptosis, alveolodental ankylosis becomes established, and the tooth will gradually be replaced by bone, still within a physiological context.

**Conclusion:**

Rhizolysis and physiological tooth resorption may occur rapidly or slowly, early or late, and this depends on the presence of the permanent tooth, or its absence due to partial anodontia.

## RESUMO

**Introdução:**

A partir de duas imagens radiográficas, revelam-se duas situações extremas distintas de reabsorção dentária fisiológica próprias dos dentes decíduos com e sem o sucessor permanente por anodontia parcial.

**Discussão:**

Todos os dentes decíduos iniciam a rizólise depois de completada a formação, graças à apoptose de suas células. Sem cementoblastos, a raiz, focalmente desnuda pela apoptose cementoblástica, inevitavelmente inicia a rizólise, que será acelerada pelos mediadores advindos do folículo pericoronário. Quando não houver o sucessor permanente por anodontia parcial, a rizólise fica muito lenta e, meses depois, sem os restos epiteliais de Malassez mortos por apoptose, a anquilose alveolodentária se estabelece e o dente será gradativamente substituído por osso, ainda dentro de um contexto fisiológico.

**Conclusão:**

A rizólise e a reabsorção dentária fisiológica podem ser rápidas ou lentas, precoces ou tardias, e isso depende da presença ou ausência do dente permanente, por anodontia parcial.

From the radiographic images, placed in the same figure for the purpose of comparison, the two final destinations and extremes of a primary tooth can be didactically illustrated, in two distinct clinical situations, dictated by the presence or absence of the permanent successor tooth. 

## THE EXTREME DESTINY OF A PRIMARY TOOTH WHEN IT HAS THE SUCCESSOR (FIG 1A)

Rhizolysis of the primary tooth begins when apical formation ends. The cementoblasts, odontoblasts, fibroblasts and epithelial cells of the epithelial rests of Malassez, randomly and gradually enter into apoptosis and denude small and multiple areas of the root surface.^1^ ^-^ ^6^.

When mineralized structures, such as bone, cement, dentin and enamel are exposed to connective tissues, they tend to attract the clasts biochemically. These are juxtaposed on the surfaces and initiate a slow process of resorption. 

When there is a source of stimulatory mediators of mineralized tissue resorption in the proximities of the denuded mineralized surface areas, the process is significantly accelerated.^7^ In primary teeth with exposed root surfaces, the sources of these mediators may be the following:

a) The pericoronal follicle of the permanent tooth, which has an important epithelial part in its structure, consisting of the reduced epithelium of the enamel organ adhered to the crown, and of the epithelial remnants of the dental lamina that formed part of the Gubernacular cord.^4^ The epithelial cells secrete a mediator denominated EGF, or epithelial or epidermal growth factor, and they biochemically command the other mediators of the pericoronal follicle. This mediator stimulates pericoronal bone resorption of teeth for the purpose of opening the way for tooth eruption to occur, moreover, it is the structure responsible for this function.^4^ ^,^ ^5^ ^,^ ^6^
b) A pulp, periapical or periodontal inflammatory process due to caries or periodontal disease in the same primary tooth in the process of rhizolysis or in a neighboring tooth.^4^

**Figure 1:**
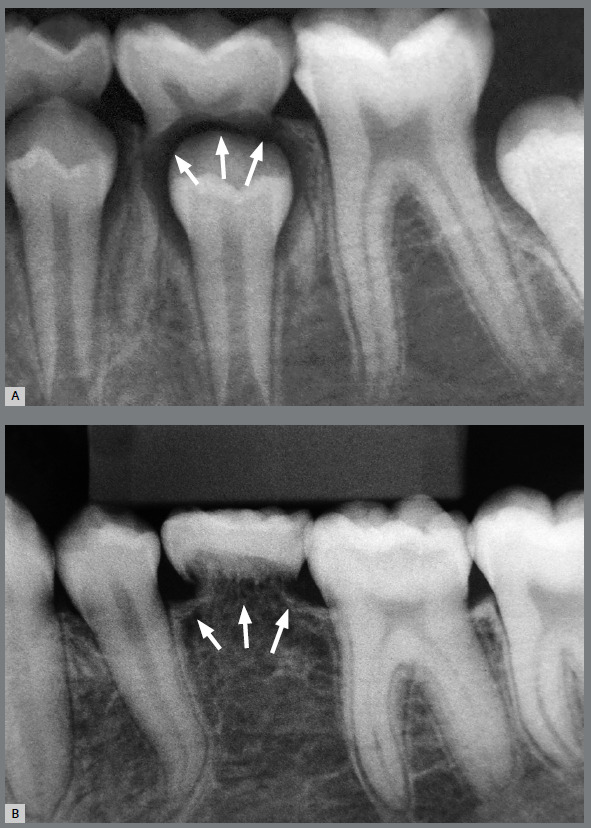
Extreme physiological resorptions, as part of the rhizolysis of primary teeth. There are two types of deciduous rhizolysis: one early and fast, as in **A**, and one late and slow, as in **B**. We emphasize that both are physiological and inevitable within the context of the exfoliation of human primary teeth.

As they approach a primary tooth, the mediators of the pericoronal follicle of the permanent tooth that stimulate bone resorption, these equally stimulate the clasts that are positioned on the denuded surface of the root.^7^ Thus rhizolysis is accelerated, especially on the surfaces facing the permanent tooth.^7^ The primary tooth gradually loses all of its periodontal and bone support, and naturally exfoliates, as this is about to occur in Figure 1A.

## THIS EXTREME DESTINY OF THE PRIMARY TOOTH OCCURS WHEN IT HAS NO PERMANENT SUCCESSOR, DUE TO PARTIAL ANODONTIA (FIG 1B)

Without the proximity of the absent permanent tooth, due to partial anodontia, there will be no acceleration of the resorption process initiated by apoptosis of the cementoblasts, odontoblasts, fibroblasts and epithelial rests of Malassez.^4^ Apoptosis affects these cells in a random manner as a biological trigger of rhizolysis, leaving areas of cement and dentin exposed in the connective tissue, which attracts the clasts to juxtapose themselves and initiate the physiological primary tooth resorption. 

Without the presence of a source of mediators that induce mineralized tissue resorption, which arise from the pericoronal follicle of the permanent tooth or an inflammatory process of pulp, periapical and/or periodontal origin, rhizolysis is extremely slow and occurs in a random and irregular manner, throughout the entire deciduous root.^4^

For many years, the primary tooth can remain with a root structure that provides periodontal support for its permanence, nevertheless, it must always be considered a tooth in a state of rhizolysis to be exfoliated. If this tooth is in a position of occlusion in an adolescent or adult patient, the masticatory load will be an occlusal trauma and will be a source of mediators that will accelerate mineralized tissue resorption through an inflammatory process induced by this cause.

Apoptosis of the epithelial rests of Malassez also eliminate this periodontal structure of primary teeth, which keep the bone distant from the root, thereby preserving the periodontal space. This function occurs due to the fact that the epithelial rests of Malassez constantly release the EGF.

Without the rests of Malassez, in a few months after the normal time of exfoliation of the primary tooth, points and foci of bone in contact with the root of the primary tooth will be observed, characterizing alveolodental ankylosis.^4^ This is to be expected and can be considered a natural physiological event in primary teeth, as part of a slower and delayed rhizolysis.

From the time of alveolodental ankylosis, in the context of normal bone remodeling in the maxilla, the clasts resorb the root structure and replace it with bone deposited by the neighboring osteoblasts. There will be a mixture between dental and bone tissues that can initially be identified in radiographic and macroscopic images. 

However, after a certain degree of development, these dental tissues will no longer be identifiable, not even macroscopically, as bone tissue will completely replace them. This may be observed, in an exemplary, didactic and elegant manner in Figure 1B. This case is self-explanatory in the image because this process that slowly follows alveolodental ankylosis is denominated “replacement” root resorption.

## FINAL CONSIDERATIONS

The two radiographic images revealed the two different extreme situations, which form part of physiological tooth resorption, in an exemplary manner: when there is a subjacent permanent successor, and when this is not present due to partial anodontia. 

In all primary teeth, rhizolysis begins after its formation is complete, thanks to the apoptosis of their cells, which represents their biological trigger. Because of this apoptosis, the teeth may exfoliate more rapidly or more slowly in the following ways:

a) When apoptosis induced by cementoblasts leaves the root denuded of these cells, the process of early rhizolysis inevitably begins. This will be accelerated by mediators arising from the pericoronal follicle. Therefore, an early and rapid rhizolysis will occur.
b) Without the mediators of the pericoronal follicle of the permanent successor tooth, rhizolysis will occur extremely slowly. Months later, without the epithelial rests of Malassez, dead due to apoptosis, alveolodental ankylosis will become established and the tooth will gradually be substituted by bone, still within a physiological context. Ankylosis and resorption by substitution constitute late and slow primary tooth rhizolysis, however, physiologically, as part of the human species, primary teeth exfoliate.

The origin of the word deciduous (primary) is a word that means “something that falls. This may occur sooner or later. Therefore, deciduous teeth are also denominated primary or temporary teeth.
